# All aboard the ChatGPT steamroller: Top 10 ways to make artificial intelligence work for healthcare professionals

**DOI:** 10.1017/ash.2023.512

**Published:** 2023-12-18

**Authors:** Lemuel R. Non

**Affiliations:** Division of Infectious Diseases, Department of Medicine, University of Iowa Hospitals and Clinics, Iowa City, IA, USA

## Abstract

Chat Generative Pre-trained Transformer (ChatGPT), the flagship generative artificial intelligence (AI) chatbot by OpenAI, is transforming many things in medicine, from healthcare and research to medical education. It is anticipated to integrate in many aspects of the medical industry, and we should brace for this inevitability and use it to our advantage. Here are proposed ways you can use ChatGPT in medicine with some specific use cases in antimicrobial stewardship and hospital epidemiology.

“Once a new technology rolls over you, if you’re not part of the steamroller, you’re part of the road.”—This was a quote by Stewart Brand, founder of *The Whole Earth Catalog* and *Wired*, about investing, but it is a perfect encapsulation of our changing technological landscape brought about by generative artificial intelligence (AI). Launched in November 2022, the Chat Generative Pre-trained Transformer (ChatGPT)^1^ is a chatbot designed to understand and generate content based on natural language processing. With its vast potential for content creation, ChatGPT, and especially its latest iteration, ChatGPT-4, has steamrolled almost every major industry, including medicine, to the extent that it is inescapable and inevitable.^2^

There exists an array of AI tools designed to enhance productivity and streamline daily work in healthcare.^3^ However, this article will focus exclusively on the capabilities of ChatGPT, presenting it as an all-encompassing AI assistant for many of the regimented tasks in antimicrobial stewardship, healthcare epidemiology, and other aspects of healthcare. ChatGPT is available for free and gives you access to ChatGPT-3.5, whereas ChatGPT-plus, the subscription-based service, offers access to the more sophisticated ChatGPT-4. Unlike ChatGPT-3.5, which handles only text-based interactions, ChatGPT-4 supports inputs and outputs, including text and images, and offers additional plugins and beta features. Throughout this article, “ChatGPT” will refer to both the free and subscription-based versions of the chatbot, with “ChatGPT-4” denoting the latter when distinctions are necessary.

Let us delve into the many ways you can be part of the steamroller and avoid the “road.” Examples of prompts used in this article are summarized in Table 1.

**Table 1.** Sample prompts for ChatGPT

What for?	Sample prompt
Copy-editing, proofreading, rewriting, and summarizing text	“Assume you’re a physician copy-editing our note, rewrite the following text in paragraphs” “Proofread this:” “Copy-edit this:” “Summarize the following text in 1 paragraph”
Creating letters and other documents	“Generate an excuse letter for a patient who is seen today in our clinic. She needs to be excused for 1 week”
Creating easy-to-understand script for communicating a medical topic	“How can I explain, in an empathic way, that prescribing antibiotics for bronchitis is not a good thing?” “Generate an easy-to-understand and compassionate script for a patient about why antibiotics are harmful in viral upper respiratory infections”
Generating content for patients	“Generate an easy-to-understand patient information on multidrug resistant bacteria in Spanish”
As a quick reference	“Does eravacycline have anaerobic coverage? Only provide responses if 100% certain” “What antibiotics can I use for Acinetobacter bacteremia? Only provide responses if 100% certain”
Creating PowerPoint slides and generating images	“Generate 2 PowerPoint slides from the following text:” “Generate a cartoon of a bacteria developing resistance to an antibiotic”
Data analysis and visualization	“Perform Chi square analysis on this dataset” “Visualize the dataset” (if you want the chatbot to determine the best graph for it) or “Visualize the dataset into bar graphs”
Medical quiz generator	“Generate 5 board exam-type questions on *Clostridium difficile*. The question should appear one at a time”
Journal club assistance	“Critique the attached article” “Summarize the paper” “What statistical analysis was used?”

Prompt Engineering Tip: Be as specific and deliberate with your instructions as much as possible. Provide examples if necessary. Note that input and output that use images or beta features (i.e. advanced data analysis, DALL-E3) require the paid ChatGPT-4 (ChatGPT-plus) subscription.

## Help with note writing

Documentation is among the burdensome tasks that healthcare providers do daily. In fact, doctors now spend between 25% and 50% of their time on documentation, contributing to burnout, dissatisfaction, and lower documentation quality.^4^ A reduction in manually entered text is found to be the most effective strategy in reducing time spent on note writing.^5^

The process of drafting comprehensive notes, replete with well-constructed sentences, impeccable grammar, spelling, and syntax, often contributes to the inefficiencies in note-writing. Here is where ChatGPT comes in. In the following section, I will expand on this feature and note several use case scenarios in which you can apply ChatGPT.

### Expanding text from medical shorthand and translating non-English language

Professionals in healthcare, irrespective of their specific roles, can leverage an AI chatbot for enhanced documentation, especially if they are familiar with medical abbreviations and shorthand. Through prompt engineering—strategically structuring textual prompts to obtain the desired output—such an AI tool can transform sentences full of shorthand into fully articulated statements.^6^ Additionally, ChatGPT has the capability to translate multilingual text to English, recognizing and converting non-English terms embedded within sentences.

### Copy-editing and proofreading text

ChatGPT can copy-edit and proofread your documentation, offer comments on your writing, and provide suggestions on how to improve your text.

### Summarizing and rewriting notes

Healthcare professionals are often tasked with summarizing texts, which may come from a variety of sources and exhibit different tones and writing styles. ChatGPT can process texts from multiple sources and either rewrite or summarize them according to your specified instructions, such as the desired number of paragraphs, tone, style, and the use of medical abbreviations. In all cases, it is imperative to avoid the use of any identifiable patient information to maintain confidentiality.

## Creating letters

Patients frequently request personalized letters from their healthcare providers. While many electronic health record systems offer letter templates, they can be cumbersome to locate and often require additional customization. ChatGPT can simplify this process by generating fully tailored letters with the appropriate prompts, circumventing the need for time-consuming template modifications. It is crucial to exclude any real patient information in these prompts to protect privacy. ChatGPT will employ placeholders (such as “Your Name,” “Patient’s Name”) in its drafts, which you can subsequently personalize using text-editing software

Other use-case scenarios in which you can enlist ChatGPT’s assistance would be in writing other professional communications. For this, it is good practice to only use the chatbot for proofreading.

## Improving patient communication

Empathy and compassion are two important things that unquestionably improve patient satisfaction, and in our daily grind of trying to meet work targets and finishing documentation these skills could be sidelined.^7^ After the launch of ChatGPT, one of the first applications of the chatbot among physicians was in enhancing bedside manners.^8^ ChatGPT can assist providers by generating empathetic, compassionate, and easy-to-understand scripts for providers to assist with communicating complex issues to patients.

## Creating easy-to-understand and customized patient content

Medical diagnoses and treatment plans are often complex for patients and their caregivers to understand in one or a few visits. We supplement this with printed handouts that are oftentimes generated from standard templates. For less common topics, providers might turn to external resources and then tailor this information to the patient’s specific needs. With the right prompts, ChatGPT can generate not only easy-to-understand content that is customized for a patient but also to translate to other languages. It is recommended, however, to thoroughly review ChatGPT’s output for accuracy and appropriateness before distribution.

## As quick reference in each step of a patient encounter

It has been demonstrated in multiple scenarios that ChatGPT can perform as well, if not better, than the average trainee in medicine in successfully taking medical exams.^9,10^ Its potential, therefore, in assisting clinicians is vast.^2^ In one study that looked at the performance of ChatGPT in answering clinical questions throughout a full clinical workflow, the authors found that the chatbot was accurate 72% of the time.^11^ You can ask it medical questions along your own workflow but always make sure to verify every response for accuracy. ChatGPT can sometimes generate inaccurate responses and make it sound true (a “hallucination”), but this happens less in ChatGPT-4. With a little bit of prompt engineering, you can further reduce hallucinations by asking it to “only provide responses if 100% certain.”

## Creating content for PowerPoint presentations and posters

**Figure 1.**
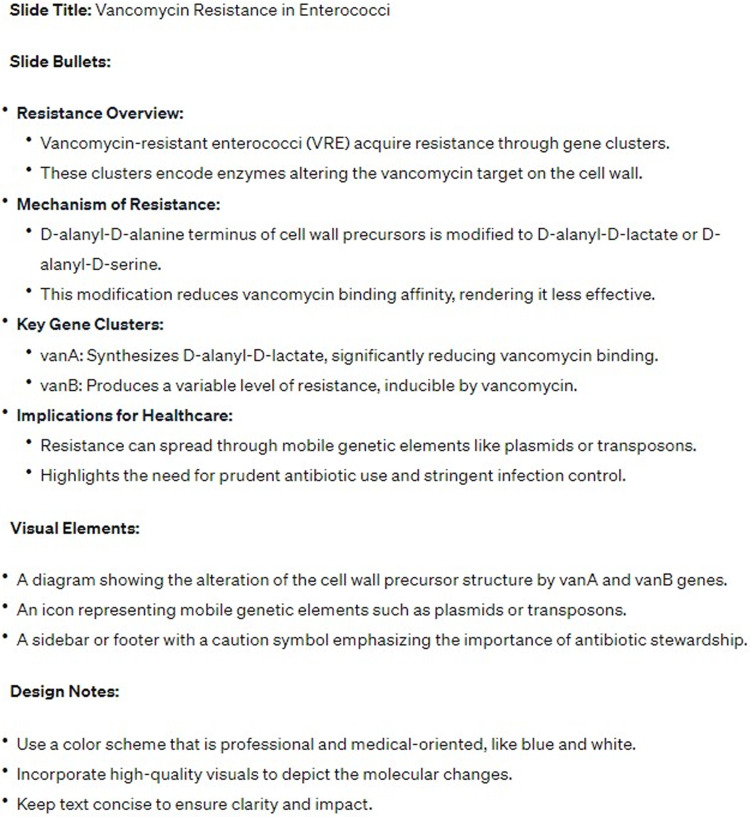
ChatGPT output showing well-formatted bulleted points about vancomycin resistance in Enterococci with suggestions regarding additional visuals and design.

Crafting PowerPoint presentations requires the ability to distill complex information into concise, well-organized bullet points. ChatGPT can streamline this process: simply provide prompts, and it will transform detailed paragraphs into presentation-ready bullet points or create new content for your slides (see Figure 1). This method is equally effective for developing content for scientific posters that use PowerPoint or a similar program.

**Figure 2.**
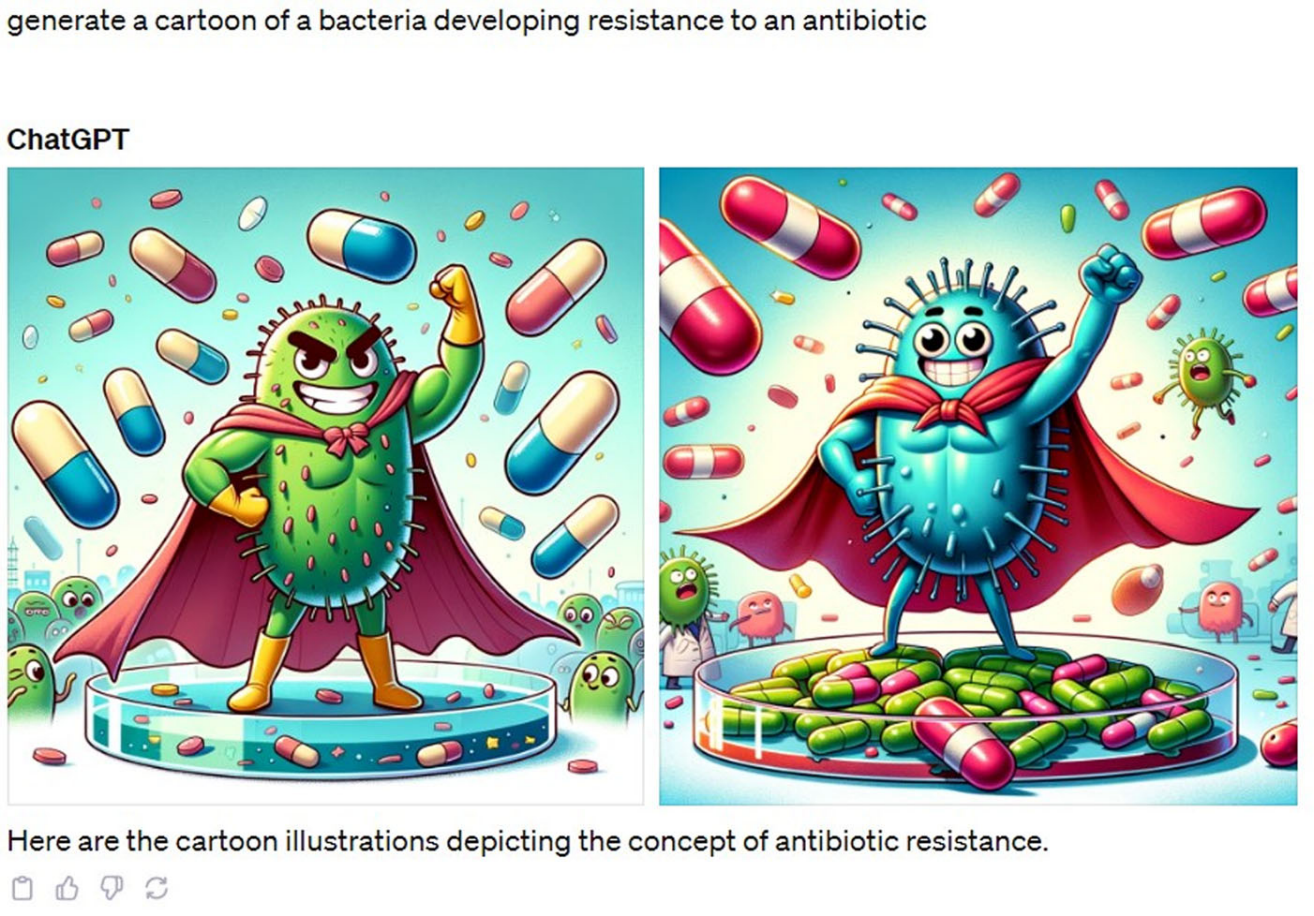
A cartoon showing a bacterium developing antibiotic resistance generated by ChatGPT-4.

Additionally, DALL-E3, an AI program by OpenAI capable of generating images from text descriptions, has been recently added to ChatGPT-4 as a beta feature.^12^ With just a few text prompts, you can create customized images that you can later add to presentations and posters (see Figure 2).

## Perform data analysis

**Figure 3.**
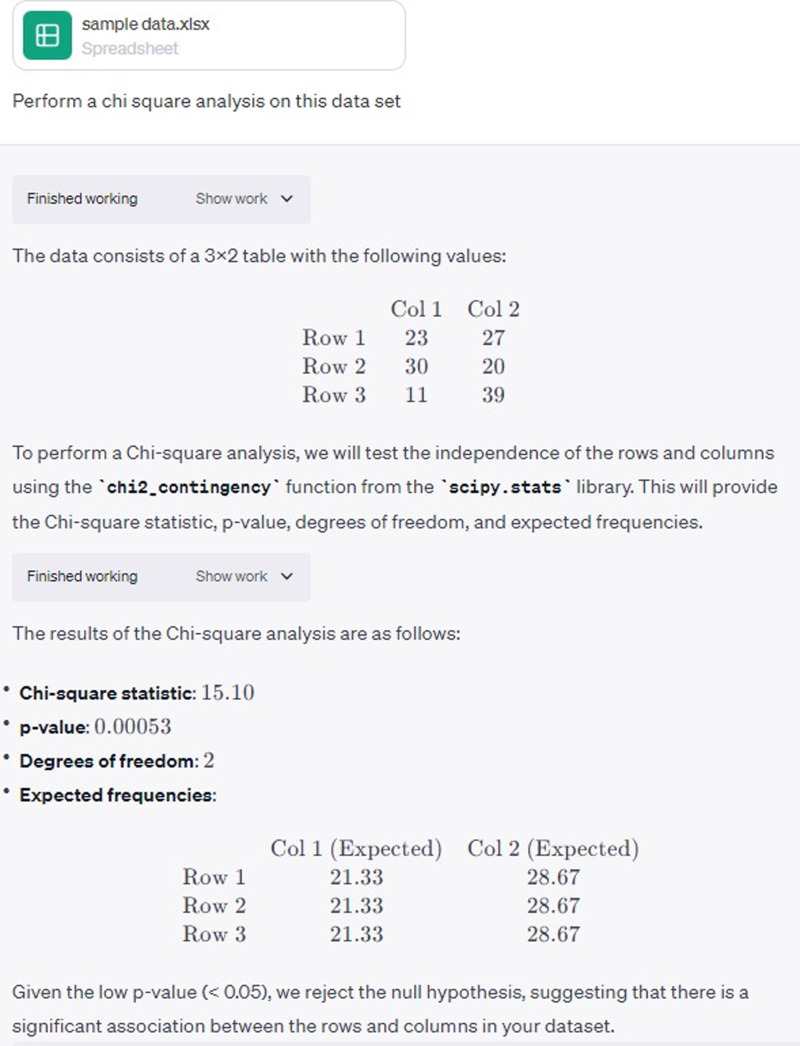
Simple prompt for asking ChatGPT-4 to perform χ^2^ analysis on an uploaded data set with output.

ChatGPT-4 recently introduced an “Advanced Data Analysis” feature, formerly known as the “code interpreter,” which can be particularly useful for those engaged in research, hospital statistics, or epidemiology. This beta feature allows users to conduct sophisticated data analysis with simple prompts (see Figure 3). This feature is not available in the free version of ChatGPT, which can sometimes hallucinate calculations.

It can also generate code with explanations for each step for common programming languages used in statistical analyses, such as R or Python. Because advanced data analysis is a beta feature of the chatbot, it is advisable to check all output for accuracy.

## Creating graphs and charts

**Figure 4.**
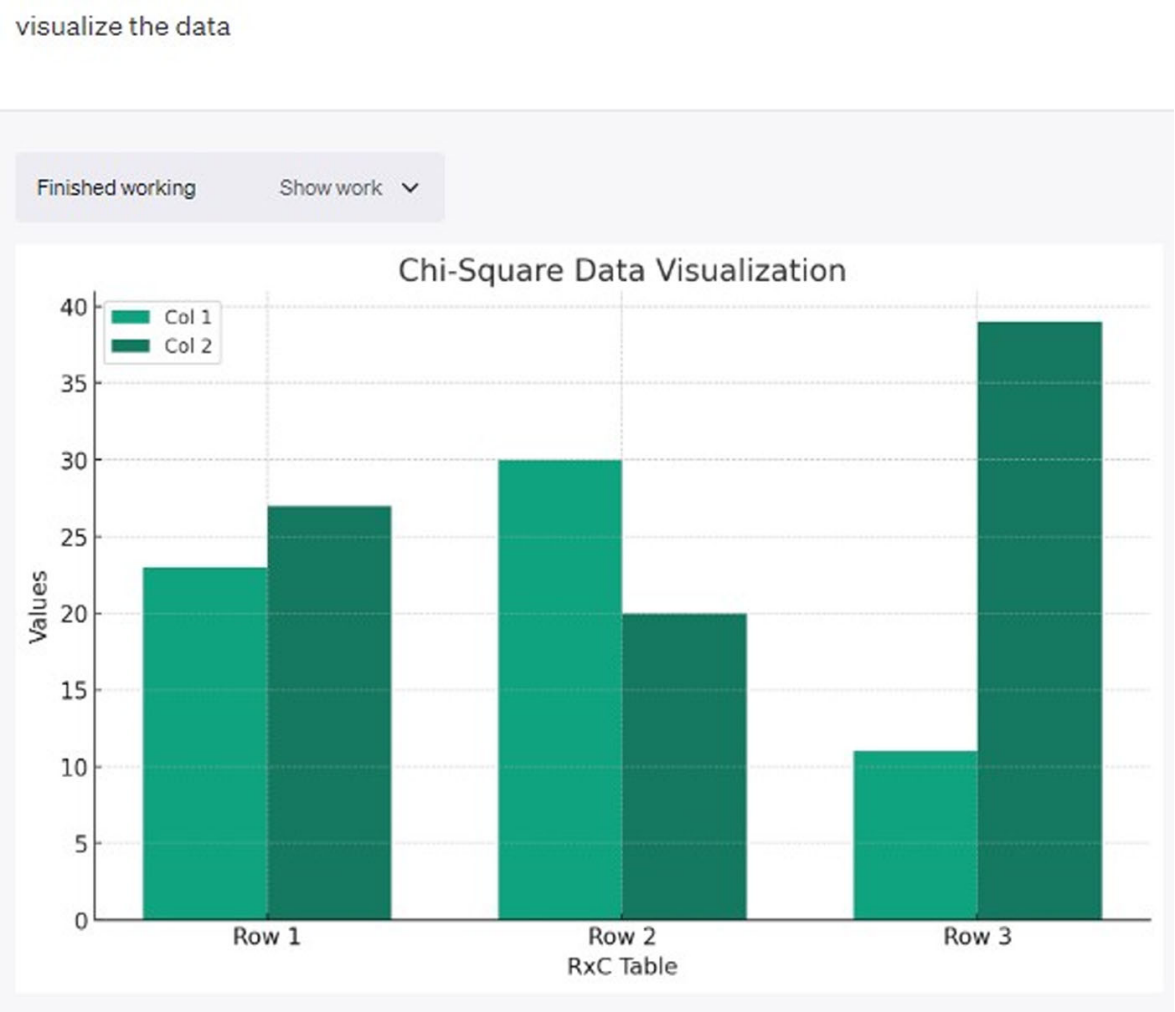
Bar graph output of the same data set used in χ^2^ analysis after asking ChatGPT-4 to “visualize” data.

The “Advanced Data Analysis” feature can also create visuals (graphs, charts, maps, etc.) for your data. You can create professional-looking visualization of your data with text prompts (see Figure 4).

## As a medical quiz generator

ChatGPT not only aces medical exams but also generates exam questions, a skillset that usually requires additional training. With some prompt engineering, you can generate test questions for diverse learners. You can also prompt ChatGPT to generate a quiz for you that will show one question at a time and provide explanations after each question is answered.

## As a journal club assistant

Lastly, one of the useful things you can do with ChatGPT is as a journal article reader. It can read a paper for you, summarize the content, answer questions about the paper, highlight the answers, and even critique the paper. I showed previously that you can do this with plug-ins^13^; however, you can do this easily with ChatGPT-4. Simply upload a document and ask your questions right away.

While the potential of ChatGPT is vast, there are limitations in its current form. The biggest concerns are data privacy and security, and inadequate regulations.^14^ Because many features of ChatGPT are still in research phase, it is good practice to avoid using identifiable health data and to follow institutional protocols regarding use of this technology.^15^ While ChatGPT is trained on large amounts of data, it may contain biases that may have negative impact on its output. The training data of the chatbot are also not current. As of this writing, the current iteration of ChatGPT-4 was trained using information up to April 2023. It is, therefore, essential to crosscheck every output of the chatbot at all times.^16^

To end this, I quote Mr. Brand again: “Technology is liberating if you make it so.”

Generative AI has the potential to improve productivity and efficiency and even add humanity back to our healthcare practice that has been taken over by corporatism. Familiarizing yourself with ChatGPT’s features and use cases, especially for many of the tasks we routinely do in antimicrobial stewardship and hospital epidemiology, can help free up some time for yourself. It might seem like a nihilistic proposition, but AI integration into health systems and in many facets of our lives is inevitable. The only way you can make this new technology work for you is by knowing how to do so.
